# Evidence that geographic variation in genetic ancestry associates with uterine fibroids

**DOI:** 10.1007/s00439-021-02322-y

**Published:** 2021-07-23

**Authors:** Jacob M. Keaton, Elizabeth A. Jasper, Jacklyn N. Hellwege, Sarah H. Jones, Eric S. Torstenson, Todd L. Edwards, Digna R. Velez Edwards

**Affiliations:** 1 Center for Precision Health Research, National Human Genome Research Institute, National Institutes of Health, Bethesda, Maryland, U.S.; 2 Division of Epidemiology, Department of Medicine, Vanderbilt University Medical Center, Nashville, Tennessee, U.S.; 3 Vanderbilt Genetics Institute, Vanderbilt University, Nashville, Tennessee, U.S.; 4 Department of Obstetrics and Gynecology, Vanderbilt University Medical Center, Nashville, Tennessee, U.S.; 5 Department of Biomedical Informatics, Vanderbilt University Medical Center, Nashville, Tennessee, U.S.; 6 Division of Genetic Medicine, Department of Medicine, Vanderbilt University Medical Center; 7 Vanderbilt Epidemiology Center, Vanderbilt University, Nashville, Tennessee, U.S.; 8 Institute for Medicine and Public Health, Vanderbilt University Medical Center, Nashville, Tennessee, U.S.

**Keywords:** leiomyoma, genetic ancestry, reproductive genetics, gynecology

## Abstract

Uterine fibroids disproportionately impact Black women. Evidence suggests Black women have earlier onset and higher cumulative risk. This risk disparity may be due an imbalance of risk alleles in one parental geographic ancestry subgroup relative to others. We investigated ancestry proportions for the 1000 Genomes phase 3 populations clustered into 6 geographic groups for association with fibroid traits in Black women (n=583 cases, 797 controls) and White women (n=1,195 cases, 1,164 controls). Global ancestry proportions were estimated using ADMIXTURE. Dichotomous (fibroids status and multiple fibroid status) and continuous outcomes (volume and largest dimension) were modeled for association with ancestry proportions using logistic and linear regression adjusting for age. Effect estimates are reported per 10% increase in genetically inferred ancestry proportion. Among AAs, West African (WAFR) ancestry was associated with fibroid risk, East African ancestry was associated with risk of multiple fibroids, Northern European (NEUR) ancestry was protective for multiple fibroids, Southern European ancestry was protective for fibroids and multiple fibroids, and South Asian (SAS) ancestry was positively associated with volume and largest dimension. In EAs, NEUR ancestry was protective for fibroids, SAS ancestry was associated with fibroid risk, and WAFR ancestry was positively associated with volume and largest dimension. These results suggest that a proportion of fibroid risk and fibroid trait racial disparities are due to genetic differences between geographic groups. Further investigation at the local ancestry and single variant levels may yield novel insights about disease architecture and genetic mechanisms underlying ethnic disparities in fibroid risk.

## INTRODUCTION

Uterine fibroids, or leiomyomata, are benign tumors of the uterus and are common among women of reproductive age(Wallach and Vlahos, 2004). Fibroid incidence increases with age ranging from 20% after menarche up to 80% by the onset of menopause(Baird et al., 2003, Cramer and Patel, 1990, Laughlin et al., 2009, Lippman et al., 2003, Marshall et al., 1997, Zimmermann et al., 2012). Fibroids are the leading indication of hysterectomy (39%) and estimates of healthcare costs range from $5.9–34.4 billion annually in the United States (Cardozo et al., 2012, Whiteman et al., 2008). Clinical and epidemiology studies have identified numerous predisposing risk factors, including obesity, age, nulliparity, family history, and race, that may play a role in the pathogenesis (Flake et al., 2003). Genetics appear to play a major role. Women with first-degree relatives with fibroids have an increased risk of developing fibroids compared to those without a family history (Sato et al., 2002, Vikhlyaeva et al., 1995). Race is the biggest risk factors for the development. Yet, the contribution of genetic ancestry to fibroid risk has been unclear.

Black women are disproportionately impacted by fibroids(Ross et al., 1986, Ryan et al., 2005). They are two to three times more likely to be diagnosed with fibroids compared to White women, and carry an increased risk for an earlier age-at-diagnosis, as well as an increased risk for larger and more numerous fibroids(Baird, Dunson, Hill, Cousins and Schectman, 2003, Kjerulff et al., 1996, Laughlin, Baird, Savitz, Herring and Hartmann, 2009, Marshall, Spiegelman, Barbieri, Goldman, Manson, Colditz, Willett and Hunter, 1997). Black women are also more likely to have a hysterectomy or myomectomy to treat fibroids(Wechter et al., 2011).

Previous studies have shown that risk of fibroproliferative disease including keloids(Niessen et al., 1999), glaucoma(Morris et al., 1999, Racette et al., 2003), hypertension(Dustan, 1992, Suthanthiran et al., 2000), nephrosclerosis(August and Suthanthiran, 2003), scleroderma(Mayes et al., 2003), sarcoidosis(Rybicki et al., 1998), asthma(Barnes et al., 2007, Lester et al., 2001, Newth et al., 2012, Nickel et al., 1999), and fibroids(Flake et al., 2003), varies by race/ethnicity. Further supporting this are findings from our group that demonstrated that the frequency of fibroproliferative risk alleles varies by geographic ancestry with a much higher burden among African-ancestry individuals and lower among European ancestry individuals(Hellwege et al., 2017). Admixture mapping analysis of fibroid risk and multiple fibroid risk also demonstrates increased risk among Black women compared to White women(Bray et al., 2017, Giri et al., 2017).

Evidence suggests that adaptive variation conferring evolutionary advantages in tropical environments inhabited by African ancestry individuals, such as connective tissue overgrowth in wound repair and hyperpigmentation as a response to ultraviolet radiation damage, may increase risk for multiple complex diseases in modern African-derived populations(Hellwege et al., 2017, Polednak, 1987). Russell et al postulated that variation protective for helminth infection may account for increased risk of fibroproliferative disease in individuals of African ancestry(Russell et al., 2015). It is unclear if genetic variation underlying fibroid risk or conferring protection against the development of fibroids has geographic origins beyond continental Africa. Defining the relationship between biogeographic ancestry and fibroid risk can provide information on the burden of genetic risk factors across ancestry groups and can illustrate differences between genetic ancestries within racial groups.

We investigated ancestry proportions for the 1000 Genomes phase 3 reference data clustered into six geographic groups with the objective of determining associations of geographically-partitioned genetic ancestry with fibroid status and fibroid traits in Black and White women from a large electronic health record (EHR) biorepository.

## MATERIALS AND METHODS

### Study Population

BioVU fibroid case and control subjects were selected as previously described(Bray, Edwards, Wellons, Jones, Hartmann and Velez Edwards, 2017, Feingold-Link et al., 2014). Briefly, The BioVU repository is a collection of stored DNA linked to de-identified EHRs at Vanderbilt University Medical Center, a resource which currently includes more than 240,000 samples for the investigation of phenotype-genotype associations(Roden et al., 2008). Fibroid cases and controls were selected from female BioVU participants over the age of 18 with at least one record of pelvic imaging. Individuals with an International Classification of Disease, ninth revision (ICD-9) diagnostic code for uterine fibroid diagnosis were selected as cases (n = 1,195 White cases, 583 Black cases), while individuals without the code, a second pelvic image, and no history of hysterectomy, myomectomy, or uterine artery embolization were selected as controls (n = 1,164 White controls, 797 Black controls). A comparison with manually reviewed records indicated a 96% positive predictive value and a 98% negative predictive value. Measurements of fibroid characteristics were manually abstracted from pelvic imaging reports and surgical reports. These characteristics include fibroid volume (n= 396 White cases, 450 Black cases), largest dimension (n = 579 White cases, 450 Black cases), and presence of multiple fibroids (i.e. single vs multiple, n = 356 White single-fibroid cases, 359 multiple-fibroid White cases, 192 Black single-fibroid cases, 258 multiple-fibroid Black cases).

### Ethical approval

The study was approved by the Institutional Review Board at Vanderbilt University Medical Center (#110407).

### SNP genotyping and quality control

Fibroid cases and controls were genotyped as previously described(Giri et al., 2017). Briefly, subjects were genotyped using the Affymetrix Axiom Biobank array (Affymetrix, Inc., Santa Clara, CA) and the Axiom World Array 3 (Affymetrix, Inc., Santa Clara, CA). DNA was purified and quantitated by PicoGreen (Invitrogen, Inc., Grand Island, NY). Standard quality control measures were applied using PLINK2(Chang et al., 2015). Sample exclusion criteria included genotypic duplicates, deviation from Hardy-Weinberg equilibrium (HWE) (p-value ≤ 1.0 × 10^−6^, and discordance between genetically-inferred sex and database sex. Closely related individuals identified by inheritance-by-descent (IBD) sharing were removed. Variants with low call rate (<95%) were excluded from subsequent analyses. Genotype data were pruned for linkage disequilibrium (LD) using a window size of 50 base pairs (bp) shifting by ten bp at an r^2^ threshold of 0.1.

1000 Genomes reference genotype data were downloaded from the UCSC server (http://hgdownload.cse.ucsc.edu/gbdb/hg19/1000Genomes/phase3/). Genotype data for 1000 Genomes samples were pruned for LD using a window size of 50 bp shifting by ten bp at an r^2^ threshold of 0.1. Variants with low call rate (<95%) were excluded from subsequent analyses. Genotype data were then randomly thinned to include 100,000 variants. For analysis of geographic ancestry proportions, LD-pruned genotype data for cases and controls were merged separately for Black and White subjects with reference genotype data. Variants with low call rate (<95%) in each merged set were excluded from subsequent analyses. Merged genotype data were then randomly thinned to include 100,000 variants.

### Assessment and cleaning of genetically-inferred reference ancestries

1000 Genomes reference samples from each geographic ancestry group (n=26) were randomly partitioned into training and testing sets. Supervised ADMIXTURE, version 1.3.0 (Alexander et al., 2009, Alexander and Lange, 2011), analysis (K=26) specifying geographic ancestry groups for each training set and estimating ancestry proportions in each testing set was used to identify heterogenous ancestry groups. Analysis showed sharing within, but not between, geographic ancestry groups corresponding to the five continental ancestries with two exceptions, sharing between African and European ancestry reference samples and sharing between East and South Asian reference samples (Supplementary Figure 1). Six 1000 Genomes reference populations were excluded from subsequent analysis due to heterogeneity. These excluded geographic ancestry groups included Americans of African Ancestry in the southwestern USA (ASW), Southern Han Chinese (CHS), British in England and Scotland (GBR), African Caribbeans in Barbados (ACB), Kinh in Ho Chi Minh City, Vietnam (KHV), and Indian Telugu from the UK (ITU) samples. Additionally, four admixed American ancestry groups (Mexican Ancestry from Los Angeles, USA [MXL], Puerto Ricans from Puerto Rico [PUR], Colombians from Medellin, Colombia [CLM], and Peruvians from Lima, Peru [PEL]) were excluded from further analysis. All excluded geographic ancestry groups, with the exception of PUR, had proportions of geographic ancestry below 60% in the testing set when compared to the corresponding geographic ancestry group training set (Supplementary Table 1).

Genotype data for 1000 Genomes samples were analyzed using ADMIXTURE(Alexander, Novembre and Lange, 2009) at several K means to determine the maximum number of ancestries that could be resolved by the software. Cross-validation error decreased for K means between one and five, stabilized at K means of five to ten, and began to increase at K means greater than 10 (Supplementary Figure 2). Subjects from remaining the 1000 Genomes populations were divided into six geographic ancestry groups. East African (EAFR) included Luhya in Webuye, Kenya (LWK) samples (n = 116). West African included Gambian in Western Divisions in the Gambia (GWD), Esan in Nigeria (ESN), Mende in Sierra Leone (MSL), and Yoruba in Ibadan, Nigeria (YRI) samples (n = 488). Northern European included Finnish in Finland (FIN) and Utah Residents (CEPH) with Northern and Western European ancestry (CEU) samples (n = 286). Southern European included Iberian individuals in Spain (IBS) and Toscani in Italia (TSI) samples (n = 269). East Asian included Chinese Dai in Xishuangbanna, China (CDX), Han Chinese in Beijing, China (CHB), and Japanese in Tokyo, Japan (JPT) samples (n = 315). South Asian included Punjabi from Lahore, Pakistan (PJL), Bengali from Bangladesh (BEB), Sri Lankan Tamil from the UK (STU), and Gujarati Indian from Houston, Texas (GIH) samples (n = 419).

### Analysis of geographic ancestry proportions in BioVU

Unsupervised ADMIXTURE analysis (K=6) of 1000 Genomes reference genotype data from each merged set (Black women and White women) was performed and ancestry proportions for each of the six reference groups were calculated (Supplementary Tables 2 and 3). These ancestry proportions were then projected onto BioVU fibroid cases and control samples in ADMIXTURE using their genotype data from the respective merged sets. Mean ancestry proportions are presented in Table 1.

### Association of geographic ancestry proportions with fibroid status and fibroid traits

Associations with global genetic ancestry proportions were computed using R, version 3.6.0 (R Core Team, 2015). Dichotomous fibroid outcomes of fibroid case/control status and single vs multiple fibroids were modeled using logistic regression against each ancestry proportion separately for Black and White subjects. Continuous fibroid traits of fibroid volume and largest fibroid dimension were modeled using linear regression against each ancestry proportion separately for Black and White subjects. Continuous outcomes were log_10_ transformed for normality. All models were adjusted for age. Additional analyses, adjusting for age and body mass index (BMI), were performed. The results for results were similar, with the exception of WAFR being a significant risk factor for volume and largest dimension in White individuals (Supplementary Table 4–7). As BMI information was missing from several women, resulting in a smaller sample size and loss of power, only age-adjusted analyses are reported here. Effect estimates are reported per 10% increase for a given inferred ancestry proportion.

## RESULTS

1000 Genomes samples were grouped in to EAFR, WAFR, NEUR, SEUR, EAS, and SAS and genetically-inferred ancestry proportions were calculated for each of these geographic groups. Ancestry proportions were then projected onto Black and White BioVU fibroid case and control subjects and tested for association with fibroid status and fibroid characteristics. These analyses included a total of 3,739 individuals from two races, Black and White. Characteristics of study participants by race (Black and White) and case/control status are presented in Table 1.

White cases were 10 years younger with marginally higher body mass index (BMI) than White controls on average. The mean age among Black participants was younger than the mean age of White participants across both cases and controls (Cases: 40.5±13.6 Black, 45.7±12.0 White, Controls: 40.4±13.5 Black, 55.6±18.9 White). Average fibroid largest dimension was marginally higher for Black cases while fibroid volume was higher among White cases. SEUR ancestry proportion was largest among White participants, while EAFR, WAFR, and EAS proportions were <5%. EAFR and WAFR ancestry proportions were largest among Black participants, while EAS and SAS proportions were <5%.

**Fig 1:**
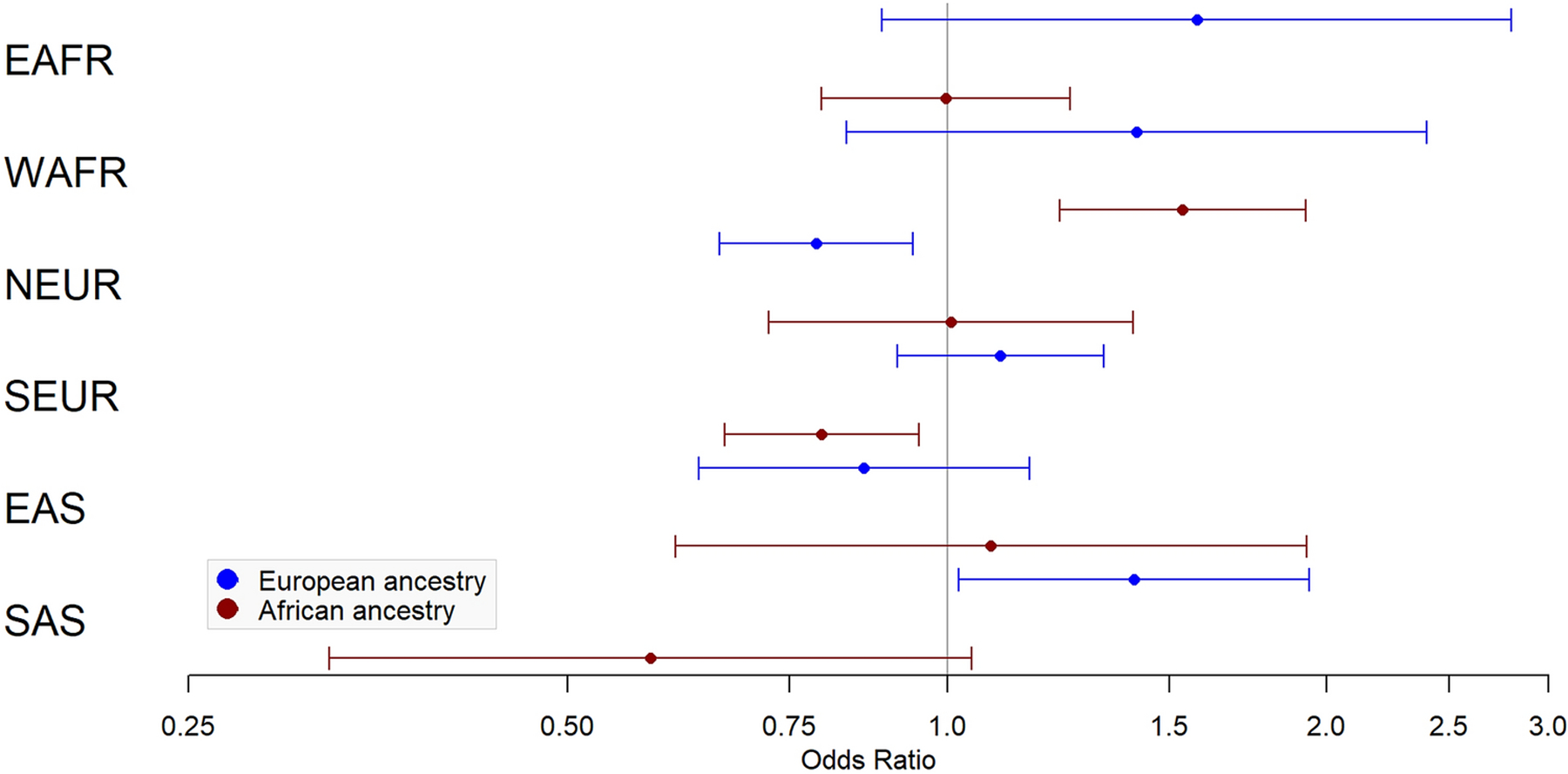
Ancestry associations with fibroid status. Forest plot of odds ratios and confidence intervals for association of fibroid status with 6 biogeographic ancestries in European ancestry (blue) and African ancestry (red). EAFR – East African; WAFR – West African; NEUR – Northern European; SEUR – Southern European; EAS – East Asian; SAS – South Asian

**Fig 2:**
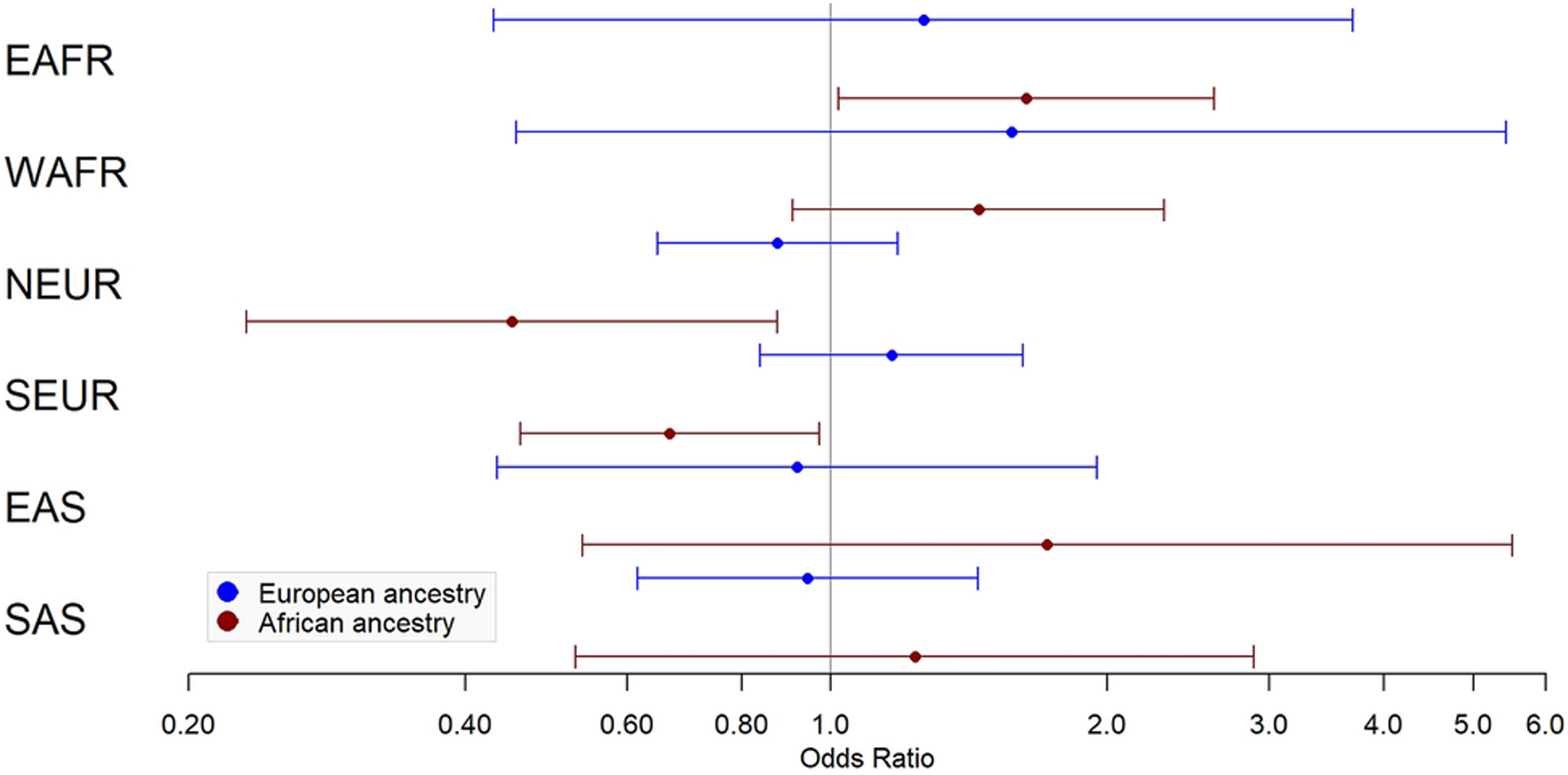
Ancestry associations with multiple fibroid status. Forest plot of odds ratios and confidence intervals for association of multiple fibroid status with 6 biogeographic ancestries in European ancestry (blue) and African ancestry (red). EAFR – East African; WAFR – West African; NEUR – Northern European; SEUR – Southern European; EAS – East Asian; SAS – South Asian

Results of ancestry proportion associations with fibroid status and multiple fibroid status are provided in Figures 1–2 and Tables 2–3. Among White subjects, every 10% higher NEUR ancestry was protective for fibroids (OR=0.79, 95% CI=0.66–0.94, *P*=8.00×10^−3^) and SAS ancestry was associated with fibroid risk (OR=1.41, 95% CI=1.02–1.94, *P*=0.04). In Black subjects, WAFR ancestry was associated with fibroid risk (OR=1.54, 95% CI=1.23–1.92, *P*=1.79×10^−4^), EAFR ancestry was associated with risk of multiple fibroids (OR=1.63, 95% CI=1.02–2.61, *P*=0.04), NEUR ancestry was protective for multiple fibroids (OR=0.45, 95% CI=0.23–0.87, *P*=0.02), and SEUR ancestry was protective for fibroids (OR=0.79, 95% CI=0.67–0.95, *P*=0.01) and multiple fibroids (OR=0.67, 95% CI=0.46–0.97, *P*=0.04).

**Fig 3:**
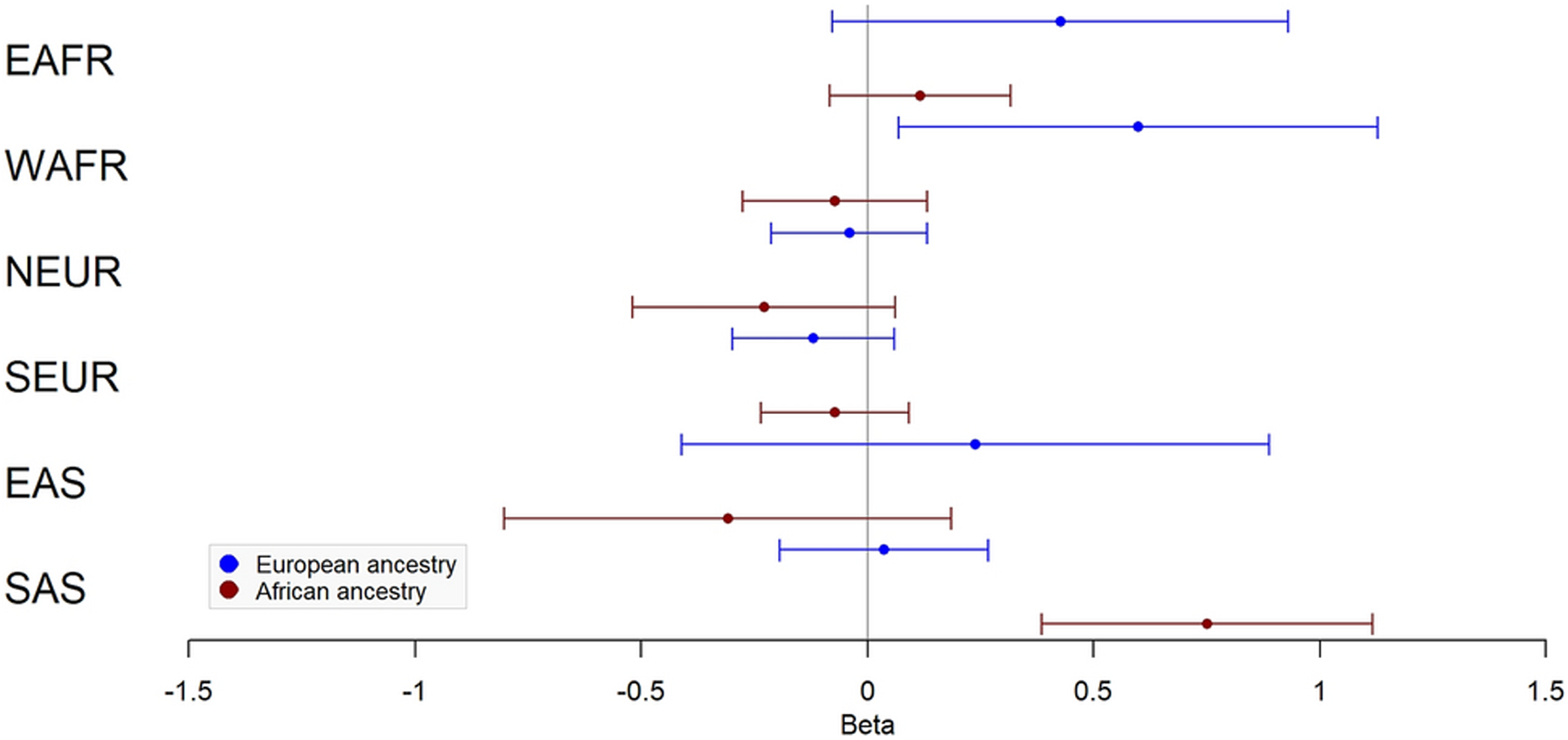
Ancestry associations with fibroid volume. Forest plot of effect and standard error for association of fibroid volume with 6 biogeographic ancestries in European ancestry (blue) and African ancestry (red). EAFR – East African; WAFR – West African; NEUR – Northern European; SEUR – Southern European; EAS – East Asian; SAS – South Asian

**Fig 4:**
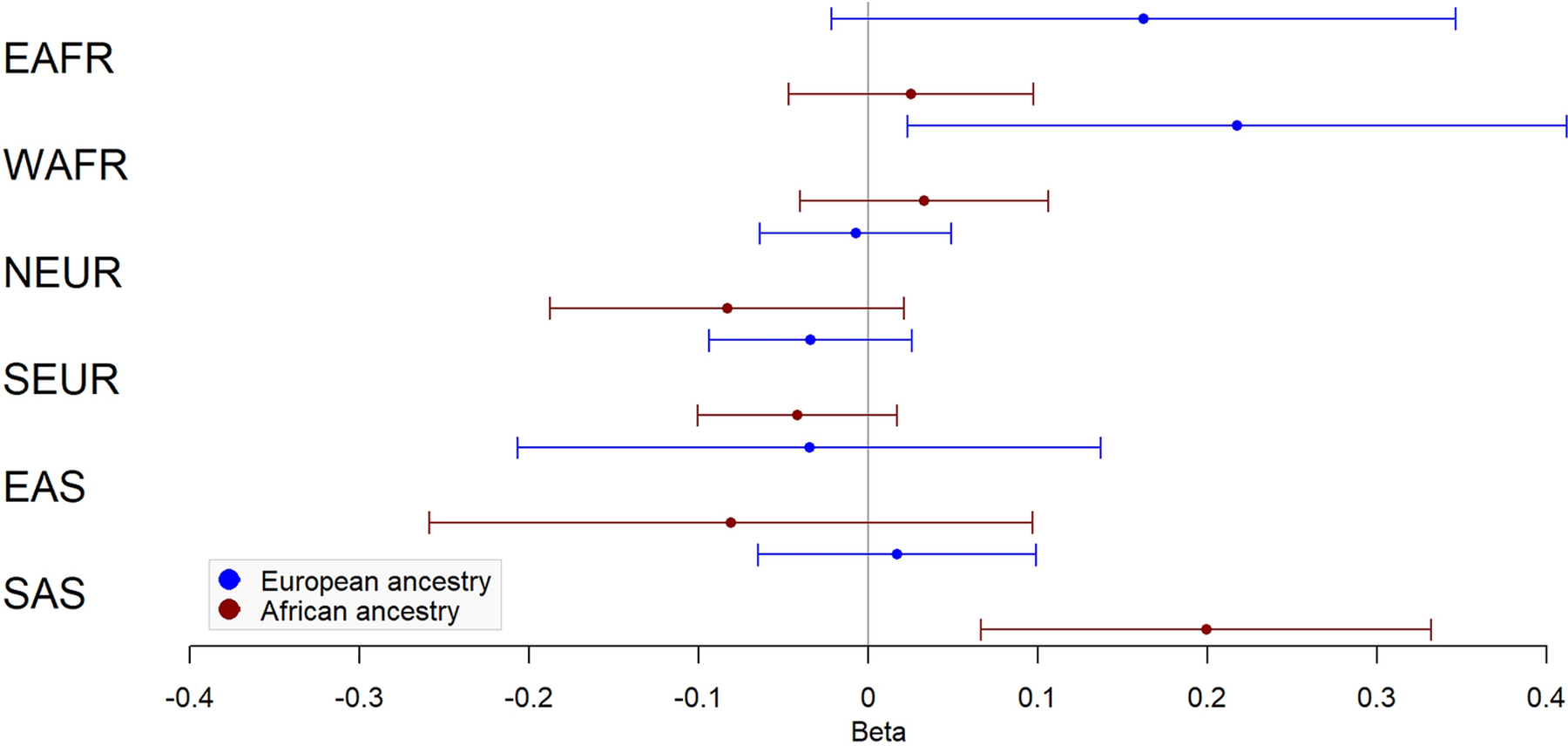
Ancestry associations with fibroid largest dimension. Forest plot of effect and standard error for association of fibroid largest dimension with six biogeographic ancestries in European ancestry (blue) and African ancestry (red). EAFR – East African; WAFR – West African; NEUR – Northern European; SEUR – Southern European; EAS – East Asian; SAS – South Asian

Results of ancestry proportion associations with fibroid characteristics are presented in Figures 3–4 and Tables 4–5. Among White subjects, WAFR ancestry was positively associated with fibroid volume (β=0.60 cubic centimeters (cm^³^), SE=0.27, *P*=0.03) and largest dimension (β=0.22 centimeters (cm), SE=0.10, *P*=0.03). In Black subjects, SAS ancestry was positively associated with fibroid volume (β=0.75 cm^³^, SE=0.19, *P*=6.73×10^−5^) and largest dimension (β=0.20 cm, SE=0.07, *P*=3.00×10^−3^). EAS ancestry was not associated with any outcome in either group.

## DISCUSSION

Previous research has focused on the association between African ancestry and fibroid risk. However, no information on which African ancestry conveyed this risk has been published or reported. Knowledge of specific African ancestry groups that confer risk would provide a more focused understanding of the geographic and biological origins of fibroids. We conducted association analyses of genetic ancestry corresponding to six biogeographic ancestries based on 1000 Genomes reference groups with fibroid status, single versus multiple fibroids, fibroid volume, and fibroid largest dimension. Our results demonstrate that fibroid risk and fibroid characteristics are influenced by genetic ancestry, with African ancestry as a risk factor for fibroids, multiple fibroids, and fibroid size, European ancestry was protective against the development of fibroids, and European ancestry was protective against the development of multiple fibroids. Previous admixture studies have reported increased fibroids risk associations with African ancestry, though these studies do not characterize ancestry proportions using a regional geographic reference inside Africa (Bray, Edwards, Wellons, Jones, Hartmann and Velez Edwards, 2017, Wise et al., 2012). The Asian ancestry proportions we observed in Black subjects are consistent with a previous study by Murray et al. examining continental ancestry proportions in Black individuals (Murray et al., 2010). A study by Richman et al. examining the association of continental ancestry proportions with lupus nephritis, another fibroproliferative disease, showed that the South Asian was the largest non-European ancestry proportion among White samples, which is consistent with our findings(Richman et al., 2012).

Two previous studies also investigated genetic ancestry and risk for fibroids. Both studies were performed exclusively in African ancestry individuals. In the Wise et al. 2013 study, European ancestry was inversely associated with risk of fibroids(Wise, Ruiz-Narvaez, Palmer, Cozier, Tandon, Patterson, Radin, Rosenberg and Reich, 2012). The authors suggested that genetic variation for fibroids differs between populations with and without African ancestry. Our study supports these results, with Northern and Southern European ancestry protective against multiple fibroids and Southern European ancestry protective against fibroids in African ancestry individuals. The other study, by Zhang et al., found similar percentages of European ancestry in cases and controls compared to the Wise et al. study; however, they failed to show a significant association between fibroids and percentage of European ancestry(Zhang et al., 2015). The lack of statistical significance in this study may be due to low power as it had a smaller sample size than both the Wise et al. and our study.

Fibroids are one of a group of diseases that vary widely in presentation but all share a disproportionate impact on individuals of African ancestry. Pathogenesis of fibroproliferative-based conditions, such as uterine fibroids, involves complex biological processes, including dysregulation of scarring and overgrowth of connective tissue (Hellwege et al., 2017, Huang and Ogawa, 2012). However, there is large heterogeneity in symptomology, fibroid location, and fibroid growth, both within and between patients, demonstrating the complexity of mechanisms underlying the development and growth of fibroids (Ciavattini et al., 2013, Commandeur et al., 2015). We have published evidence that polygenic selection has occurred at risk loci for several fibroproliferative traits between African and non-African populations, which may contribute to racial disparities in risk and severity (Hellwege et al., 2017). In these studies we demonstrated that across published GWAS of fibroproliferative diseases there is strong evidence of increasing selection among those of African ancestry when compared to those of non-African ancestry. It may be that fibroid risk alleles have pleiotropic effects on diseases (share common genetic risk factors) and this is the cause of the observed racial disparity in fibroproliferative diseases.

More research is needed in this area, as this study possesses limitations that must be addressed. The cohort from which the study population was obtained was well defined, as all women in the cohort all had pelvic imaging. Case status was based on a single ICD-9 code for fibroids. ICD codes are largely used for billing purposes and not specifically designed for research purposes. Reliance on these codes may lead to bias in results due to misclassification. However, a portion of the data was independently validated through manual chart abstraction. With the strong performance of the fibroid phenotype classification algorithm, the possibility of results being due to misclassification of the outcome is unlikely. While there is significant heritability for fibroids, environmental and lifestyle factors also play a role. Future studies should extend this investigation by looking at the role of non-genetic risk factors and their potential interaction with genetic ancestry. Finally, a replication cohort was unavailable for this study. Replication of this research, with a larger sample size and increased power, would also aid in validation of these findings.

Although racial disparities are well-documented, this study is unique in showing evidence of association of genetically-inferred geographic ancestry with fibroid status and fibroid traits and establishes that a portion of fibroid trait racial disparities are due to genetic differences between groups with varying ancestral geographic origins. Further investigation at the local ancestry and single variant levels may yield novel insights about disease architecture and genetic mechanisms underlying racial disparities in fibroid risk. Together, these analyses may provide insight into the geographic factors underlying the origin of fibroid risk variants.

## Supplementary Material

1729163_Sup_Tabfig

## Figures and Tables

**Table 1.** Demographics of the study participants

Characteristic mean (±SD)	White Cases (N = 1,195)	White Controls (N = 1,164)	Black Cases (N = 583)	Black Controls (N = 797)
**Age, years**	45.7 (±12.0)	55.6 (±18.9)	40.5 (±13.6)	40.4 (±13.5)
**BMI, kg/m** ^**2**^	29.1 (±7.7)	28.0 (±7.2)	28.1 (±15.3)	28.1 (±15.3)
**Fibroid number, n (%)**				
** 1**	356 (49.79%)		192 (42.67%)	
** >1**	359 (50.21%)		258 (57.33%)	
**Fibroid largest dimension, cm**	3.85 (±2.74)	-	3.99 (±2.90)	-
**Fibroid volume, cm** ^**3**^	56.5 (±122.4)	-	48.2 (±139.2)	-
**Genetic ancestry proportions mean (**±**SD)**				
** EAFR**	0.01 (±0.02)	0.01 (±0.01)	0.38 (±0.04)	0.38 (±0.05)
** WAFR**	0.01 (±0.02)	0.00 (±0.02)	0.35 (±0.04)	0.34 (±0.05)
** NEUR**	0.25 (±0.06)	0.26 (±0.04)	0.07 (±0.03)	0.07 (±0.03)
** SEUR**	0.60 (±0.05)	0.60 (±0.04)	0.12 (±0.05)	0.13 (±0.07)
** EAS**	0.03 (±0.02)	0.03 (±0.03)	0.04 (±0.02)	0.04 (±0.02)
** SAS**	0.11 (±0.04)	0.10 (±0.02)	0.04 (±0.02)	0.04 (±0.02)

SD – standard deviation; kg/m^2^ – kilograms per meter squared; cm – centimeters; EAFR – East African; WAFR – West African; NEUR – Northern European; SEUR – Southern European; EAS – East Asian; SAS – South Asian

**Table 2.** Ancestry associations with dichotomous fibroid traits in White individuals

	Fibroid Status (Cases = 1,195, Controls = 1,164)		Multiple Fibroids (Multiple = 359, Single = 356)	
	OR (95% CI)	P-value	OR (95% CI)	P-value
**EAFR**	1.58 (0.89–2.80)	1.20×10^−1^	1.26 (0.43–3.70)	6.73×10^−1^
**WAFR**	1.41 (0.83–2.40)	2.00×10^−1^	1.57 (0.45–5.43)	4.75×10^−1^
**NEUR**	**0.79 (0.66–0.94)**	**8.00×10** ^**−3**^	0.87 (0.65–1.18)	3.84×10^−1^
**SEUR**	1.10 (0.91–1.33)	3.14×10^−1^	1.16 (0.84–1.62)	3.66×10^−1^
**EAS**	0.86 (0.63–1.16)	3.24×10^−1^	0.92 (0.43–1.95)	8.25×10^−1^
**SAS**	**1.41 (1.02–1.94)**	**3.70×10** ^**−2**^	0.94 (0.62–1.44)	7.89×10^−1^

OR – odds ratio; CI – confidence interval; EAFR – East African; WAFR – West African; NEUR – Northern European; SEUR – Southern European; EAS – East Asian; SAS – South Asian. Significant associations shown in bold.

**Table 3.** Ancestry associations with dichotomous fibroid traits in Black individuals

	Fibroid Status (Cases = 583, Controls = 797)		Multiple Fibroids (Multiple = 258, Single =192)	
	OR (95% CI)	P-value	OR (95% CI)	P-value
**EAFR**	1.00 (0.79–1.25)	9.82×10^−1^	**1.63 (1.02–2.61)**	**4.20×10** ^**−2**^
**WAFR**	**1.54 (1.23–1.92)**	**1.79×10** ^**−4**^	1.45 (0.91–2.30)	1.19×10^−1^
**NEUR**	1.01 (0.72–1.40)	9.68×10^−1^	**0.45 (0.23–0.87)**	**1.80×10** ^**−2**^
**SEUR**	**0.79 (0.67–0.95)**	**1.10×10** ^**−2**^	**0.67 (0.46–0.97)**	**3.50×10** ^**−2**^
**EAS**	1.08 (0.61–1.93)	7.87×10^−1^	1.72 (0.54–5.52)	3.62×10^−1^
**SAS**	0.58 (0.32–1.05)	7.10×10^−1^	1.23 (0.53–2.89)	6.28×10^−1^

OR – odds ratio; CI – confidence interval; EAFR – East African; WAFR – West African; NEUR – Northern European; SEUR – Southern European; EAS – East Asian; SAS – South Asian. Significant associations shown in bold.

**Table 4.** Ancestry associations with continuous fibroid traits in White individuals

	Volume (N = 396)		Largest Dimension (N = 579)	
	BETA (SE)	P-value	BETA (SE)	P-value
**EAFR**	0.43 (0.26)	9.80×10^−2^	0.16 (0.09)	8.40×10^−2^
**WAFR**	**0.60 (0.27)**	**2.80×10** ^**−2**^	**0.22 (0.10)**	**2.90×10** ^**−2**^
**NEUR**	−0.04 (0.09)	6.52×10^−1^	−0.01 (0.03)	7.96×10^−1^
**SEUR**	−0.12 (0.09)	1.94×10^−1^	−0.03 (0.03)	2.66×10^−1^
**EAS**	0.24 (0.33)	4.71×10^−1^	−0.03 (0.09)	6.92×10^−1^
**SAS**	0.04 (0.12)	7.55×10^−1^	0.02 (0.04)	6.86×10^−1^

BETA – effect; SE – standard err; EAFR – East African; WAFR – West African; NEUR – Northern European; SEUR – Southern European; EAS – East Asian; SAS – South Asian. Significant associations shown in bold.

**Table 5.** Ancestry associations with continuous fibroid traits in Black individuals

	Volume (N = 450)		Largest Dimension (N = 450)	
	BETA (SE)	P-value	BETA (SE)	P-value
**EAFR**	0.12 (0.10)	2.54×10^−1^	0.03 (0.04)	4.90×10^−1^
**WAFR**	−0.07 (0.10)	4.94×10^−1^	0.03 (0.04)	3.77×10^−1^
**NEUR**	−0.23 (0.15)	1.24×10^−1^	−0.08 (0.05)	1.19×10^−1^
**SEUR**	−0.07 (0.08)	3.93×10^−1^	−0.04 (0.03)	1.65×10^−1^
**EAS**	−0.31 (0.25)	2.23×10^−1^	−0.08 (0.09)	3.73×10^−1^
**SAS**	**0.75 (0.19)**	**6.73×10** ^**−5**^	**0.20 (0.07)**	**3.00×10** ^**−3**^

BETA – effect; SE – standard err; EAFR – East African; WAFR – West African; NEUR – Northern European; SEUR – Southern European; EAS – East Asian; SAS – South Asian. Significant associations shown in bold.

## Data Availability

The data underlying this article will be shared on reasonable request to the corresponding author.
