# Supplementary material for: Evidence that geographic variation in genetic ancestry associates with uterine fibroids
Source: Hum Genet. Author manuscript; Available in PMC 2022 Oct 1. (PMC8463481; doi:10.1007/s00439-021-02322-y)
Supplement: 1729163_Sup_Tabfig [file NIHMS1729163-supplement-1729163_Sup_Tabfig.docx]

**Supplementary Tables**

**Supplementary Table 1. Admixture proportions by 1000 Genomes population**

| **Population** | **Ancestry Proportion** |
| --- | --- |
| GIH | 60.24% |
| CEU | 70.30% |
| PJL | 73.55% |
| CHB | 77.45% |
| YRI | 82.87% |
| BEB | 83.79% |
| IBS | 84.84% |
| STU | 85.80% |
| CDX | 87.67% |
| MSL | 91.35% |
| TSI | 92.07% |
| FIN | 92.43% |
| JPT | 92.91% |
| ESN | 94.46% |
| LWK | 94.69% |
| GWD | 96.66% |

LWK – Luhya in Webuye, Kenya; GWD – Gambian in Western Divisions in the Gambia; ESN – Esan in Nigeria; MSL – Mende in Sierra Leone; YRI – Yoruba in Ibadan, Nigeria; FIN – Finnish in Finland; CEU – Utah Residents (CEPH) with Northern and Western European Ancestry; IBS – Iberian Population in Spain; TSI – Toscani in Italia; CDX – Chinese Dai in Xishuangbanna, China; CHB – Han Chinese in Beijing, China; JPT – Japanese in Tokyo, Japan; PJL – Punjabi from Lahore, Pakistan; BEB – Bengali from Bangladesh; STU – Sri Lankan Tamil from the UK; GIH – Gujarati Indian from Houston.

**Supplementary Table 2. Admixture proportions in 1000 Genomes reference samples using BioVU White SNP set with k=6**

|  | **EAFR** | **WAFR** | **NEUR** | **SEUR** | **EAS** | **SAS** |
| --- | --- | --- | --- | --- | --- | --- |
| **Q1** | 0.95 | 0.33 | 0.00 | 0.00 | 0.00 | 0.00 |
| **Q2** | 0.01 | 0.67 | 0.00 | 0.00 | 0.00 | 0.00 |
| **Q3** | 0.00 | 0.00 | 0.65 | 0.02 | 0.00 | 0.02 |
| **Q4** | 0.00 | 0.00 | 0.35 | 0.97 | 0.00 | 0.04 |
| **Q5** | 0.01 | 0.00 | 0.00 | 0.00 | 1.00 | 0.04 |
| **Q6** | 0.03 | 0.00 | 0.00 | 0.01 | 0.00 | 0.90 |

EAFR – East African; WAFR – West African; NEUR – Northern European; SEUR – Southern European; EAS – East Asian; SAS – South Asian.

**Supplementary Table 3. Admixture proportions in 1000 Genomes reference samples using BioVU Black SNP set with k=6**

|  | **EAFR** | **WAFR** | **NEUR** | **SEUR** | **EAS** | **SAS** |
| --- | --- | --- | --- | --- | --- | --- |
| **Q1** | 0.95 | 0.36 | 0.00 | 0.00 | 0.00 | 0.00 |
| **Q2** | 0.00 | 0.64 | 0.00 | 0.00 | 0.00 | 0.00 |
| **Q3** | 0.00 | 0.00 | 0.65 | 0.03 | 0.00 | 0.03 |
| **Q4** | 0.01 | 0.00 | 0.35 | 0.95 | 0.00 | 0.04 |
| **Q5** | 0.01 | 0.00 | 0.00 | 0.00 | 1.00 | 0.04 |
| **Q6** | 0.03 | 0.00 | 0.00 | 0.02 | 0.00 | 0.89 |

EAFR – East African; WAFR – West African; NEUR – Northern European; SEUR – Southern European; EAS – East Asian; SAS – South Asian; Q1-6 – Ancestry proportions from ADMIXTURE.

**Supplementary Table 4. Ancestry associations, adjusted for age and body mass index, with dichotomous fibroid traits in White individuals**

|  | **Fibroid Status**  **(Cases = 1,082, Controls = 1,131)** | | **Multiple Fibroids**  **(Multiple = 354, Single = 347)** | |
| --- | --- | --- | --- | --- |
|  | **OR (95% CI)** | **P-value** | **OR (95% CI)** | **P-value** |
| **EAFR** | 1.73 (0.86-3.49) | 1.26x10⁻¹ | 0.91 (0.27-3.01) | 8.72x10⁻¹ |
| **WAFR** | 1.43 (0.77-2.66) | 2.59x10⁻¹ | 1.35 (0.37-4.88) | 6.51x10⁻¹ |
| **NEUR** | **0.79 (0.66-0.95)** | **1.30x10⁻²** | 0.88 (0.65-1.19) | 4.03x10⁻¹ |
| **SEUR** | 1.09 (0.89-1.33) | 4.03x10⁻¹ | 1.26 (0.89-1.79) | 1.92x10⁻¹ |
| **EAS** | 0.84 (0.62-1.15) | 2.78x10⁻¹ | 0.80 (0.35-1.82) | 5.93x10⁻¹ |
| **SAS** | **1.51 (1.07-2.12)** | **1.80x10⁻²** | 0.94 (0.61-1.44) | 7.81x10⁻¹ |

OR – odds ratio; CI – confidence interval; EAFR – East African; WAFR – West African; NEUR – Northern European; SEUR – Southern European; EAS – East Asian; SAS – South Asian. Significant associations shown in bold.

**Supplementary Table 5. Ancestry associations, adjusted for age and body mass index, with dichotomous fibroid traits in Black individuals**

|  | **Fibroid Status**  **(Cases = 544, Controls = 687)** | | **Multiple Fibroids**  **(Multiple = 249, Single =179)** | |
| --- | --- | --- | --- | --- |
|  | **OR (95% CI)** | **P-value** | **OR (95% CI)** | **P-value** |
| **EAFR** | 1.06 (0.83 -1.35) | 6.38x10⁻¹ | **1.72 (1.06-2.78)** | **2.80x10⁻²** |
| **WAFR** | **1.49 (1.17-1.90)** | **1.00x10⁻⁴** | 1.62 (0.98-2.69) | 6.20x10⁻² |
| **NEUR** | 0.90 (0.63-1.29) | 5.74x10⁻¹ | **0.44 (0.22-0.86)** | **1.70x10⁻²** |
| **SEUR** | **0.79 (0.65-0.96)** | **1.60x10⁻²** | **0.60 (0.40-0.89)** | **1.20x10⁻²** |
| **EAS** | 1.10 (0.61-1.99) | 7.51x10⁻¹ | 1.72 (0.52-5.65) | 3.73x10⁻¹ |
| **SAS** | 0.73 (0.39-1.36) | 3.20x10⁻¹ | 1.25 (0.52-3.02) | 6.19x10⁻¹ |

OR – odds ratio; CI – confidence interval; EAFR – East African; WAFR – West African; NEUR – Northern European; SEUR – Southern European; EAS – East Asian; SAS – South Asian. Significant associations shown in bold.

**Supplementary Table 6. Ancestry associations, adjusted for age and body mass index, with continuous fibroid traits in White individuals**

|  | **Volume**  **(N = 388)** | | **Largest Dimension**  **(N = 570)** | |
| --- | --- | --- | --- | --- |
|  | **BETA (SE)** | **P-value** | **BETA (SE)** | **P-value** |
| **EAFR** | 0.27 (0.29) | 3.52x10⁻¹ | 0.09 (0.11) | 3.94x10⁻¹ |
| **WAFR** | 0.49 (0.30) | 1.01x10⁻¹ | 0.16 (0.11) | 1.46x10⁻¹ |
| **NEUR** | -0.05 (0.09) | 6.09x10⁻¹ | -0.01 (0.03) | 7.11x10⁻¹ |
| **SEUR** | -0.11 (0.10) | 2.75x10⁻¹ | -0.02 (0.03) | 4.62x10⁻¹ |
| **EAS** | 0.57 (0.41) | 1.66x10⁻¹ | -0.03 (0.10) | 7.86x10⁻¹ |
| **SAS** | 0.07 (0.12) | 5.57x10⁻¹ | 0.03 (0.04) | 4.68x10⁻¹ |

BETA – effect; SE – standard err; EAFR – East African; WAFR – West African; NEUR – Northern European; SEUR – Southern European; EAS – East Asian; SAS – South Asian. Significant associations shown in bold.

**Supplementary Table 7. Ancestry associations, adjusted for age and body mass index, with continuous fibroid traits in Black individuals**

|  | **Volume**  **(N = 427)** | | **Largest Dimension**  **(N = 427)** | |
| --- | --- | --- | --- | --- |
|  | **BETA (SE)** | **P-value** | **BETA (SE)** | **P-value** |
| **EAFR** | 0.13 (0.10) | 1.98x10⁻¹ | 0.03 (0.04) | 4.60x10⁻¹ |
| **WAFR** | -0.05 (0.11) | 6.36x10⁻¹ | 0.05 (0.04) | 1.96x10⁻¹ |
| **NEUR** | -0.25 (0.15) | 9.50x10⁻² | -0.09 (0.05) | 9.20x10⁻² |
| **SEUR** | -0.11 (0.09) | 2.29x10⁻¹ | -0.06 (0.03) | 8.10x10⁻² |
| **EAS** | -0.24 (0.26) | 3.49x10⁻¹ | -0.05 (0.09) | 5.68x10⁻¹ |
| **SAS** | **0.75 (0.19)** | **1.01x10⁻⁴** | **0.19 (0.07)** | **6.00x10⁻³** |

BETA – effect; SE – standard err; EAFR – East African; WAFR – West African; NEUR – Northern European; SEUR – Southern European; EAS – East Asian; SAS – South Asian. Significant associations shown in bold.

**Supplementary Figures**

**Supplementary Figure 1. Estimation of 26 ancestries from 1000 Genomes reference data.** Stacked bar plot of ancestry proportions estimated in ADMIXTURE for 26 1000 Genomes populations grouped by continent. ACB – African Caribbeans in Barbados; ASW – African Ancestry in southwestern United States of America; ESN – Esan in Nigeria; GWD – Gambian in Western Divisions in the Gambia; LWK – Luhya in Webuye, Kenya; MSL – Mende in Sierra Leone; YRI – Yoruba in Ibadan, Nigeria; CLM – Colombians from Medellin, Colombia; MXL – Mexican Ancestry from Los Angeles, United States of America; PEL – Peruvians from Lima, Peru; PUR – Puerto Ricans from Puerto Rico; CDX – Chinese Dai in Xishuangbanna, China; CHB – Han Chinese in Beijing, China; CHS – Southern Han Chinese ; JPT – Japanese in Tokyo, Japan; KHV – Kinh in Ho Chi Minh City, Vietnam; CEU – Utah Residents (CEPH) with Northern and Western European Ancestry; FIN – Finnish in Finland; GBR – British in England and Scotland; IBS – Iberian Population in Spain; TSI – Toscani in Italia; BEB – Bengali from Bangladesh; GIH – Gujarati Indian from Houston; ITU – Indian Telugu from the United Kingdom; PJL – Punjabi from Lahore, Pakistan; STU – Sri Lankan Tamil from the UK


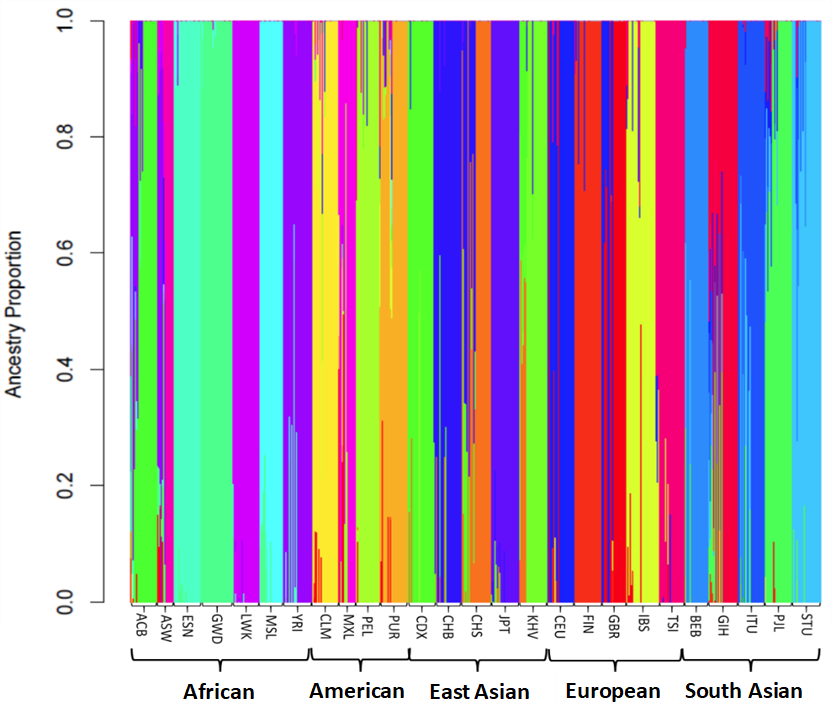


**Supplementary Figure 2. Analysis of optimal K means.** Plot of cross-validation errors at kmeans ranging from 1 to 20.

**
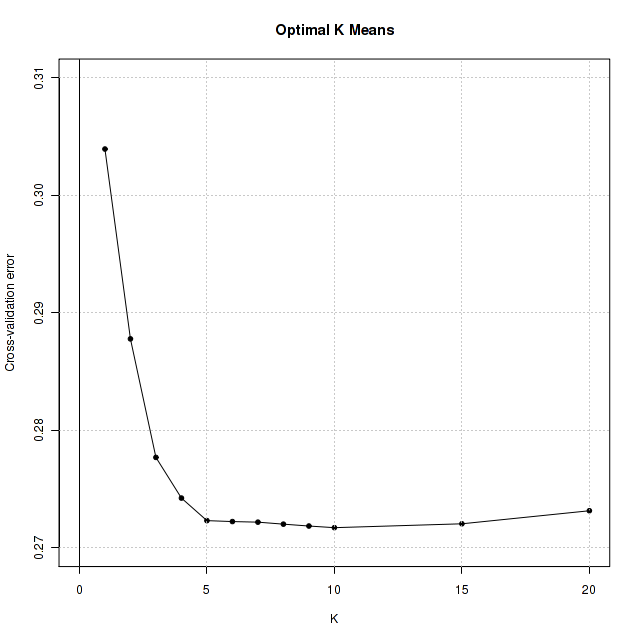
**
